# Is the Biopesticide from Tea Tree Oil an Effective and Low-Risk Alternative to Chemical Pesticides? A Critical Review

**DOI:** 10.3390/molecules29143248

**Published:** 2024-07-09

**Authors:** Magdalena Dziągwa-Becker, Marta Oleszek

**Affiliations:** 1 Department of Weed Science and Tillage Systems, Institute of Soil Science and Plant Cultivation, State Research Institute, Wrocław ul. Orzechowa 61, 50-540 Wrocław, Poland; 2 Department of Biochemistry and Crop Quality, Institute of Soil Science and Plant Cultivation, State Research Institute, Czartoryskich 8, 24-100 Puławy, Poland; moleszek@iung.pulawy.pl

**Keywords:** tea tree oil, biopesticides, low-risk pesticides, fungicide, tea tree extract

## Abstract

The use of chemical pesticides in agriculture contributes to soil, water and air pollution, biodiversity loss, and injury to non-target species. The European Commission has already established a Harmonized Risk Indicator to quantify the progress in reducing the risks linked to pesticides. Therefore, there is an increasing need to promote biopesticides, or so-called low-risk pesticides (LRP). Tea tree oil (TTO) is known for its antiseptic, antimicrobial, antiviral, antifungal, and anti-inflammatory properties. TTO has been extensively studied in pest management as well as in the pharmaceutical and cosmetic industry; there are already products based on its active substances on the market. This review focuses on the overall evaluation of TTO in terms of effectiveness and safety as a biopesticide for the first time. The collected data can be an added value for further evaluation of TTO in terms of the authorization extension as a fungicide in 2026.

## 1. Introduction

The use of chemical pesticides in agriculture can lead to soil, water and air pollution, loss of biodiversity, and injury to non-target organisms. The European Commission has already developed a Harmonized Risk Indicator to quantify the progress in reducing the risks related to pesticides. This represents a 20% reduction in risk from pesticide use over the past five years. The European Commission plans to reduce the overall use and risk of chemical pesticides by 50% by 2030, which will lead to incorporating a number of steps. This will revise the Sustainable Use of Pesticides Directive, enhance provisions on integrated pest management (IPM), and promote safe and alternative ways of protecting harvests from pests and diseases. The Commission will also simplify the registration of pesticides containing biologically active substances and facilitate the environmental risk assessment of pesticides, which will act to reduce the length of the pesticide authorization process by Member States [1]. Therefore, there is a strong need for harmonization of requirements for the efficacy evaluation of low-risk plant protection products to facilitate their placement on the market. The efficacy evaluation may be flexible regarding the variability or level of effectiveness and less supporting efficacy data may be needed [2]. In light of the above, there is an increasing need to promote biopesticides or so-called low-risk pesticides (LRP). For example, botanical pesticides, comprising plant essential oils (PEOs) and extract from different parts of plants, act in a variety of ways against pests such as insects, fungi, bacteria, nematodes, and plant host cells infected with viral pathogens [3]. 

A well-known group of plant-derived essential oils called terpenoids are highly concentrated hydrophobic complex mixtures obtained from different parts of plants such as flowers, fruits, leaves, or peels. Examples of essential oils are tea tree oil (TTO), lemon oil, thyme essential oil, lavender oil, clove oil, or lemongrass oil [4].

*Melaleuca alternifolia*—a tea tree native to Australia, New Zealand, and some parts of Asia is known for its antiseptic, antifungal, antimicrobial, antiviral, and anti-inflammatory properties. TTO is produced by steam distillation of the leaves and terminal branchlets of *M. alternifolia*. There are a number of studies on TTOs favourable properties in the field of pest management as well as in the pharmaceutical and cosmetic industry; products based on components of TTO are on offer [5]. TTO is a complex mixture made up of fifteen main components, with four major terpenoids: terpinen-4-ol (30–48%), γ-terpinene (10–28%), α-terpinene (5–13%), and 1.8%-cineole (0.1–15%). Their content varies significantly depending on the harvest period and geolocalisation. The majority of the active substances forming TTO are volatile or highly volatile. The composition of TTO changes particularly in the presence of atmospheric oxygen, but also when the oil is exposed to light and higher temperatures [6]. Extract from the tea tree was approved in 2009 in the EU under Regulation 1107/2009/EC as a fungicide, is only authorized for use in the greenhouse, and the expiration of approval finishes in 2026. The representative formulation is “Timorex Gold^®^”, containing 660 g/kg of TTO. It is a water-soluble concentrate with a surface mode of action, leading to the destruction of the cells’ integrity, respiration inhibition, and ion transport process [7,8,9,10].

In terms of their functions, pesticides can be classified as herbicides, fungicides, algicides, rodenticides, and so on. However, according to their sources, pesticides are classified as synthetic pesticides, biopesticides, or low-risk pesticides (LRP). In general, biopesticides are cheap, environmentally-friendly, specific in their mode of action, sustainable, should not leave residues, and are not associated with the release of greenhouse gases [3]. TTO represents a plant-based biopesticide.

To be classified as low-risk, a pesticide must meet the regular approval criteria defined in article 22(3) of Regulation EC No 1107/2009, as well as additional criteria specified in Annex II, point 5 of this regulation, amended by Commission Regulation (EU) 2017/1432 [11,12]. To assess compliance with these requirements, the substance must undergo various tests, which often pose difficulties for applicants and prevent a full assessment. 

This review is an attempt to evaluate the TTO in terms of meeting the above-mentioned requirements and being designated as a low-risk pesticide. The latest literature was reviewed concerning the application in agriculture, efficacy against plant pathogens, and safety for humans, other organisms, and the environment. Web of Science, Scopus, and Google Scholar databases have been searched with keywords: ‘tea tree oil’, ‘TTO’, ‘*Melaleuca alternifolia* oil’, ‘low-risk pesticides’, ‘biopesticides’, ‘risk assessment’, ‘herbicides’, ‘fungicide’ and combination of these. Based on the above, conclusions were drawn regarding TTO’s potential to be considered a low-risk pesticide.

## 2. Extract from Tea Tree as a Biopesticide

The literature is replete with studies demonstrating the remarkable performance of TTO as an antimicrobial, mainly antifungal, antibacterial, and antiviral, but also a pesticidal agent [8,9,10,13]. According to document No. SANTE/11312/2021 V2, tea tree extract represents difficult or unique commodities, which means that difficult commodities should only be fully validated if they are frequently analysed. If they are only analysed occasionally, validation may be reduced to just checking the reporting limits using spiked blank extracts; therefore, the analytical procedure is simplified [14].

TTO proved to be efficient at a concentration of 0.9 g/L against *Botritis cinerea* and *Rhizopus stolonifera*, fungi causing disease in strawberries during storage. It has been revealed that the mechanism of action is not only via direct interaction with the fungus itself but also via defensive responses of fruit tissue [15]. Another mechanism was proposed by Wang et al. [16], who stated that TTO affects *B. cinerea* mitochondria function through the inhibition of the tricarboxylic acid cycle, pyruvate metabolism, amino acid metabolism, and membrane-related pathways in mitochondria, as well as the promotion of sphingolipid metabolism, accelerating cell death.

Antifungal activity of TTO has also been demonstrated against *Aspergillus niger* in grapes, with terpene-4-ol, α-terpineol, and 3-carene identified as its main components [17]. Complete inhibition of fungal growth was detected at concentrations ≥ 3.6 µL/mL during the 5 days of incubation. The mode of action is based on the inhibition of mycelium growth and spore germination mainly by terpene-4-ol and α-terpineol.

TTO also works well as an addition to traditional fungicides. Reuveni et al. [18] conducted research using a hybrid fungicide containing TTO and difenoconazole for grape powdery mildew in seven field trials and two large-scale demonstration trials in two different regions—Chile and Israel. Foliar sprays of difenoconazole-TTO were applied as a preventive treatment in field trials at 40–80 up to 80–160 g/ha active ingredient, and they were highly effective in controlling powdery mildew on the fruit clusters of both wine and table grapes in experimental and large-scale demonstration trials. This provided up to 99% efficacy in disease incidence and severity compared with the untreated control. Difenoconazole-TTO was more effective than other DMI fungicides, including difenoconazole, a pre-mixed fungicide boscalid-pyraclostrobin, treatments that included various fungicides applied in rotation, or mixtures of fungicides. The results suggest that a combination of difenoconazole and TTO with a reduced synthetic chemical load can be included in powdery mildew control programs for grapevine as a strategic approach in fungicide resistance management in vineyards. 

The insecticidal potential of TTO formulations was tested for contact and stomach poison toxicities against various stages of the *Spodoptera littoralis* larvae under laboratory conditions by Şimşek et al. [19]. In the contact toxicity test, the formulations were tested at different stages of larvae by topical application. Taking contact toxicity into account among the tested formulations, pure TTO F14 (formulation with terpinen-4-ol) and F15 (formulation with monoterpenes from TTO) caused the highest mortality in the *S. littoralis* 3rd stage larvae after 72 h, amounting to 91.72% and 89.20% mortality, respectively. The authors have also shown that two other formulations, namely F16 (containing linalool) and F17 (containing eugenol) caused a stomach poisoning effect. These results revealed that TTO and its main components containing formulations have the potential to control the lepidopteran pest species. Additionally, the study shows that TTO and the formulations produced toxic effects on *S. littoralis* larvae in different ways.

Choi et al. [20] evaluated the toxicity of 53 plant essential oils for their insecticidal activities against eggs, nymphs, and adults of *Trialeurodes vaporariorum* without direct contact. The experiment revealed that TTO was highly effective against *T. vaporariorum* adults, nymphs, and eggs at 0.0023, 0.0093, and 0.0047 µL/mL air, respectively. The mortality was 91 ± 4.6%, 97 ± 3.1%, and 88 ± 6.1%, respectively. It revealed that the mode of delivery of these essential oils was largely a result of action in the vapor phase. Moreover, TTO significantly inhibited three enzymes in the cereal weevil, *Sitophilus zeamais*: glutathione S-transferase (GST), carboxylesterase (CarE), and acetylcholinesterase (AChE) [21]. The LC_50_ of TTO was 6.78 mg/L. The insecticidal activity of TTO is due to its direct action on the hydrogen carrier, blocking the flow of electrons and interfering with the synthesis of the respiratory chain of the mitochondria [9]. TTO also has anti-oviposition activity, which has been reported in previous studies. For example, Benelli et al. [22] has shown the effectiveness of TTO against *Ceratitis capitata* and *Psyttaliaconcolor*, two species of Mediterranean fruit fly. In the contact assay, the LC_50_ was 0.117 μL oil/cm^2^ and 0.147 μL oil/cm^2^, and in the fumigation assay, the LC50 was 2.239 μL oil/L air and 9.348 μL oil/L air for *C. capitata* and *P. concolor,* respectively. It was also reported that TTO has a repellent activity against *Culex pipiens pallens* (Diptera: Culicidae) and larvicidal activity against *Culex quinquefasciatus* (Diptera: Culicidae) although the effect was much lower than other tested essential oils [23]. The repellent effect of TTO has also been tested on leaf-cutting ants. It turned out that TTO was effective at concentrations of 1 and 10%, but the effect lasted only for four days and was evident at short distances of less than 1 cm. Nonetheless, TTO is considered a promising short-term repellent for protecting attractive food from leaf-cutting ant attacks [24]. Lin et al. [25] evaluated the removal efficacy of pesticide residues from cowpeas using different concentrations of TTO. The objective pesticide residues were detected using GC-MS. The results showed that TTO was able to remove three kinds of pesticides from cowpeas. Moreover, the removal efficacy increased with increasing concentrations of TTO [25]. 

## 3. Safety of Tea Tree Oil as a Biopesticide

The toxicity of TTO to humans has been widely tested due to its application in cosmetics and medicine [26]. This is also an important issue when we consider TTO as a biopesticide due to the safety of farmers and other users. No less important is the assessment of the safety of TTO for the natural environment, including the soil, water, and various organisms living there.

### 3.1. Human Safety

#### 3.1.1. Carcinogenicity and Mutagenicity

In accordance with current knowledge, TTO is not mutagenic, but some cytotoxic effects on human epithelial and fibroblast cells were reported at a concentration of 300 μg/mL. The concentration of 100 μg/mL was proven not to be cytotoxic. The values of relative cytotoxicity (NR_50_), which is the concentration needed to induce a 50% growth reduction, were estimated at 550 and 450 μg/mL for fibroblast and epithelial cells, respectively. It is worth mentioning that such concentrations of TTO in human blood are very unlikely to be reached. TTO is sparingly soluble in water [27].

#### 3.1.2. Sensitizing Properties

Hausen et al. [28] have performed experiments on guinea pigs where they were sensitized by a modified FCA method with freshly distilled TTO, oxidized TTO, the monoterpene and sesquiterpene fraction, and 1, 8-cineole. They have proven that fresh TTO is a very weak sensitizing material, whereas oxidized TTO is three times stronger when used topically on the skin. Moreover, the monoterpene fraction is a stronger sensitizer than the sesquiterpene fraction. These must be considered a danger for users of this biopesticide, which may be responsible for the development of allergic contact dermatitis. The patch test studies proved that 1.6% of people have some allergic reaction to TTO [27]. However, it is known that TTO is easily degraded when repeatedly exposed to air, light, and high temperatures, causing the formation of peroxides and degradation products. The strongest skin sensitizer is 1,2,4-trihydroxymenthane. To reduce the formation of these oxidation products, manufacturers consider the use of antioxidants or specific packaging. In rabbits, no toxic effects were observed after dermal application of up to 2 g per kg body weight. Moreover, as reported by the Scientific Committee on Consumer Products (SCCP), safety of processing and storage of TTO can be achieved by the control of p-cymene content [29].

#### 3.1.3. Toxicity

Despite the sensitization issue, there are also many reports about harmfulness after consumption. The LD_50_ (the amount of a chemical that is lethal to one half (50%) of the experimental animals exposed) in rats has been stated to be 1900 mg/kg. For comparison, table salt’s LD_50_ is 3000 mg/kg. Thus, the TTO dose which is harmful seems to be quite high. Nonetheless, due to reported cases of poisoning, diarrhea, and even short-term coma, undiluted TTO should not be taken orally and is classified as harmful via the oral route for humans according to the European Union’s Dangerous Preparations Directive [30].

### 3.2. Environmental Safety

#### 3.2.1. Ecotoxicology

As the oil is obtained from the leaves of the *Melaleuca alternifolia* plant, it is a completely natural product. Nonetheless, previous research proves that TTO is highly toxic to aquatic invertebrates. It was found that TTO is toxic to non-target organisms (NTO) such as *Daphnia magna*, which shares the same ecological niche as *Aedes albopictus*, which TTO was applied against. TTO also had a remarkable acute toxicity towards adults of the non-target arthropod *D. magna*, with a LC_50_ = 80.636 ppm [31]. The Committee of Risk Assessment (RAC) reported in its newest opinion that the half maximal effective concentration (EC_50_) for *D. magna* was 0.591 mg/L and toxicity for fish, invertebrates (one chronic toxicity study on the effects of TTO on *Chironomus riparius* is available and leads to a NOEC of 4.36 mg/L and a corresponding EC_50_ of 28.3 mg/L), algae, and the higher aquatic plants has been proven. According to these results, RAC concludes that TTO should be classified in the category of aquatic acute (H400) [32]. Moreover, Braga et al. [33] reported that TTO is extremely toxic to predators, which play an important role in the biological control of pests. According to this, TTO should not be used as an insecticide in association with biological control using *Podisus nigrispinus*.

Nonetheless, according to EFSA reports, TTO is not phytotoxic and significant exposure of TTO to birds is not expected due to volatilization and rapid degradation of the active ingredient in the environment [34]. There is some inconsistency with RAC reports; therefore, more research is needed to obtain comprehensive data.

The influence of TTO on the soil fauna has been tested on *Folsomia candida*, a standard species for ecotoxicological tests [35]. No negative effects have been reported. 

To manage the problems with potential toxicity, volatility and insolubility, as well as to increase efficiency, nanoparticles can be used. The advantages of this technology consist of slow, gradual, and controlled release of the product. New methods of preparation, such as microencapsulation, prevent immediate contact of TTO with the environment and ensure controlled release, which increases the safety of the environment [36].

#### 3.2.2. Persistence and Biodegradability

In light of the listed requirements for biopesticide characteristics, biodegradation seems to be one of the crucial traits. With regard to the persistence of TTO during storage, constituents of TTO undergo photooxidation within a few days to several months, depending on the storage method [28]. Unfortunately, it leads to degradation products, such as peroxides, epoxides, and endoperoxides, for example, ascaridol and 1,2,4-trihydroxymethane, which are moderate to strong sensitizers. As was reported by Hausen et al. [28], in the oxidized oil, the p-cymene content increased dramatically from about 2.0% to 11.5%, and peroxide content increased from less than 50 ppm to greater than 500 ppm within two months. Nonetheless, TTO indeed degrades rapidly in the environment. Up to 90% of TTO has been shown to volatilize within 24 h after application and residue studies indicate the lack of detectable residues of three of the major constituents of TTO at 48 h post-application, so biodegradability can be taken for granted. Moreover, residues of the constituents of TTOin drinking water are not expected when pesticide products are used according to label instructions. There is a lack of information on the natural background levels of TTO in soil, water and sediments and biopesticides with TTO are not directly applied to water; therefore, residues of TTO in drinking water are unlikely to occur [34,37]. On the other hand, the parameters such as half-life in soil and bioaccumulation factor (BCF) were not established for TTO itself, but only for selected components. Moreover, according to the RAC, TTO should be considered as having a high potential to bioaccumulate, because 12 of the 15 of its known constituents have exceeded permissible values of octanol-water partition coefficient (log K_ow_) and 6 of the 12 do not meet the limit for BCF [32].

#### 3.2.3. Effectiveness

Low-risk substances need to exhibit recognized efficacy. Effectiveness should normally be evaluated under conditions that replicate the practical use of the product; this means, in general, evaluation of trials under field or glasshouse conditions. However, additional data from carefully designed small-scale laboratory and growth chamber studies may form a vital component of the overall information package provided to support authorization. Laboratory studies provide data on the mode of action, the susceptibility of target pests or hosts, including different life stages (where appropriate), dose-response behaviour and the effect of environmental, agronomic, and other factors on the product. Appropriately conducted studies provide key supporting information which supports the subsequent number of larger-scale (including GEP) field studies required and assist in the interpretation of trial data [2]. Some field experiments with TTO have been successfully conducted. Reuveni et al. [38] tested Timorex Gold, a preparation based on TTO, against black Sigatoka in bananas and powdery mildew in cucumbers. The mode of action was disruption of the fungal cell membrane and cell wall, as well as shrinkage and disruption of fungal hyphae and conidial cells, respectively. The antifungal activity of Timorex Gold has also been tested against *Alternaria* sp. leaf spot of Chinese cabbage, downy mildew in lettuce cultivation [39,40]. Moreover, greenhouse assays have been conducted with pots planted with 10 pre-germinated seeds of pepper and infected by *Pythiumaphanidermatum*. In this experiment, Timorex gold exhibited the lowest activity among all tested preparations, but it was still significantly higher in comparison with the negative control. The EC_50_ was 175.33 mg/L and 35%, in the in vitro and greenhouse tests, respectively [41]. 

Another field experiment was conducted on *Nicotiana glutinosa*. The tobacco mosaic virus was successfully mitigated by sprays of TTO at concentrations of 100, 250, and 500 ppm. Subsequently, a reduction in lesion development was observed at least 10 days post-application [42].

#### 3.2.4. Resistance

EPPO Standard PP 1/213 Resistance risk analysis indicates which information should be provided to indicate whether resistance is likely to occur during the practical use of the low-risk product. Resistance may be of less relevance for substances with multiple modes of action or pheromones. Many existing resistance management approaches (e.g., alternation) are appropriate or can be adapted for strategies for use with low-risk plant protection products [2]. The studies on the resistance of a microorganism to TTO are still in progress. Until now, it has been proven that some species of bacteria are more resistant to TTO and demand higher doses than others. For example, *Pseudomonas aeruginosa* has shown lower susceptibility to TTO, with concentrations of 2–8% required to inhibit it, in comparison to 0.06–0.5% in the case of most tested bacteria [43]. The reason for low susceptibility is that the outer membrane is more difficult to permeate for TTO. According to the authors, it is unlikely that resistance to TTO would develop in P. aeruginosa following long-term continuous exposure. The development of resistance in other species of bacteria also seems unlikely to occur. To the best of our knowledge, no studies are proving that bacteria acquire resistance over time as a result of long-term use of TTO.

Dalio et al. [9] raised questions regarding the ability of TTO to perform as a resistance inducer. This was examined by TTO application to banana plants challenged by *Fusarium oxysporum* f. sp. *cubense* (Foc) causing Fusarium wilt, as well as to tomato plants challenged by Xanthomonas campestris. The results demonstrated that TTO is an efficient resistance inducer since it enhances the expression of marker genes in banana and tomato plants for both systemic acquired resistance (SAR) and induced systemic resistance (ISR) pathways. The authors revealed that TTO sprayed on field-grown banana plants infected with Foc and greenhouse tomato plants infected with Xanthomonas campestris resulted in resistance induction in both hosts [9].

### 3.3. Risk Assessment

To assess the potential of TTO to be recognized as a low-risk pesticide, it is necessary to consider the eligibility criteria included in Annex II point 5 of Regulation (EC) 1107/2009 (Table 1). Moreover, under Regulation (EC) No 1272/2008, low-risk candidates must not be classified as any of the following hazard statements: H200, H201, H202, H203, H300, H301, H310, H311, H317, H330, H331, H334, H350, H351, H340, H341, H360, EUH070, H370, H371, H400, H410 [44,45]). Furthermore, such pesticides should not be identified as priority substances under Directive No 2000/60/EC [46] or as endocrine disruptors, neurotoxins, or immunotoxins. 

There is no basis to conclude that TTO is a carcinogenic or mutagenic substance, but there is still insufficient data for a proper evaluation. With regard to the toxicity of TTO in reproduction, RAC concluded that no classification of TTO is needed for germ cell mutagenicity based on the negative results in in vitro tests on bacteria and mammalian cells, as well as in vivo mouse micronucleus tests. On the other hand, RAC proposed the classification of TTO as toxic to fertility because of the results of the studies on rats and dogs, which proved that TTO decreased sperm count and mobility [32].

Moreover, TTO is considered a sensitizing and irritative chemical for the skin. Much evidence has been recorded that TTO is toxic when ingested. According to safe sheets of TTO [47], it should not enter the environment due to its toxicity, and its vapor may be explosive. On the contrary, in the latest opinion, RAC denies the explosive characteristics of TTO because the oxygen balance of its ingredients is well below the trigger of −200 [32]. 

Kim et al. [48] showed the inhibitory action of water-soluble tea tree extract on steel corrosion in a hydrochloric acid solution. Inhibitory performance was tested on non-passivated mild steel (MS) exhibiting uniform corrosion and passivated stainless steel (STS) exhibiting pitting corrosion. Particularly, tea tree extract has a corrosion inhibitory effect that has never been reported, and it was selected as a green organic inhibitor because of its availability, economical price, and good antioxidant properties. Additionally, according to RAC, TTO does not have the properties of substances corrosive to metals such as acidic or basic functional groups, halogens, or the ability to form complexes with metals [32].

Hawkins et al. [49] tried to find the relationship between lavender and TTO and pediatric endocrine disorders. This study did not find evidence to support the claim that TTO is related to endocrine disruption in children. Because this potential link remains a concern among pediatric care providers and parents, epidemiological research to address the proposed link is needed [49]. Cases of TTO toxicosis have been reported in dogs and cats following dermal application for therapeutic reasons. Typical signs of neurotoxicity were observed, such as depression, weakness, incoordination, ataxia, and muscle tremors [6]. 

With regard to persistence, there is not enough data for its full assessment because half-life in soil and BCF were not established for TTO itself, but only for selected ingredients. Nonetheless, the values of BCF and log K_ow_ for most ingredients exceed the permitted limit of 500 and 4, respectively.

Among the prohibited hazard statements mentioned above, TTO is associated with H317 and H400, which are hazards of skin sensitization, and aquatic acute, respectively. Nonetheless, TTO is not listed as a priority substance under Directive No 2000/60/EC, but is listed on the United States Toxic Substances Control Act (TSCA). The criteria and their fulfilment for TTO, stated based on the present literature review, are summarised in Table 1.

## 4. Conclusions

To conclude, although TTO is undoubtedly toxic when ingested in higher doses, no unreasonable adverse effect to the population will result from the use of TTO as a pesticide according to the label instructions. TTO should not be phytotoxic due to rapid degradation of the active substance and a significant exposure to birds is not expected; however, there is too little data on that matter. TTO is a more eco-friendly, plant-origin, sustainable, biodegradable, and cost-effective alternative to chemical pesticides. Among a long list of advantages, there are also some drawbacks that we cannot forget. TTO is highly toxic to aquatic invertebrates and toxic to NTOs, whereas it is not acutely toxic to fish. TTO can probably control only one pest at a time, and there might be issues with the quality of the material due to inconsistencies in the concentration of the active ingredient from different geographical locations and harvest periods. Another issue could be connected with the short shelf life of TTO formulations, which on the one hand serves as an advantage because it does not remain in the environment but on the other hand, it protects the crops only for a short time. The physical characteristics of TTO present certain difficulties for the formulation and packaging of products. Its lipophilicity leads to miscibility problems in water-based products, while its volatility means that packaging must provide an adequate barrier to volatilization. Therefore, more research is required for the development of low-risk formulations with traits enabling them to compete with synthetic pesticides and to check their effectiveness not only under controlled conditions but on the field as well. Almost 10% of known PEOs have commercial applications [50]. Biopesticides also obtained from other essential oils like lavender essential oil [51], thyme oil, or sweet orange oil have vast potential [52]. The mutual use of LRP and chemical pesticides could be a reasonable research direction in the forthcoming years.

## Figures and Tables

**Table 1:** Eligible criteria for low-risk pesticides included in Annex II, point 5 of Regulation (EC) 1107/2009, and their fulfilment for TTO.

Exclusion Criteria for Low-Risk Pesticide	Fulfilment for TTO
carcinogenicity	yes
mutagenicity	yes
toxic to reproduction	no
very toxic or toxic	no
sensitising chemicals	no
explosive	yes
corrosive	yes
endocrine disrupter	yes
neurotoxicity	no
immunotoxic effects	no
persistent (half-life in soil is more than 60 days)	not established
bioconcentration factor is higher than 100	not established

## Data Availability

No new data were created.
